# Plant species richness increases with light availability, but not variability, in temperate forests understorey

**DOI:** 10.1186/s12898-020-00311-9

**Published:** 2020-07-29

**Authors:** Carsten F. Dormann, Maurizio Bagnara, Steffen Boch, Judith Hinderling, Andrea Janeiro-Otero, Deborah Schäfer, Peter Schall, Florian Hartig

**Affiliations:** 1 Biometry & Environmental System Analysis, University of Freiburg, Tennenbacher Str. 4, 79104 Freiburg, Germany; 2 Senckenberg Biodiversity and Climate Research Centre (SBiK-F), Senckenberganlage 25, 60325 Frankfurt am Main, Germany; 3 WSL Swiss Federal Research Institute, Zürcherstrasse 111, 8903 Birmensdorf, Switzerland; 4 Institut of Plant Sciences, University of Bern, Altenbergrain 21, 3013 Bern, Switzerland; 5 Silviculture and Forest Ecology of the Temperate Zones, University of Göttingen, Büsgenweg 1, 37077 Göttingen, Germany; 6 Theoretical Ecology, University of Regensburg, Universitätsstraße 31, 93053 Regensburg, Germany

**Keywords:** Understorey vegetation, Temperate forest, Species richness, Light availability

## Abstract

**Background:**

Temperate forest understorey vegetation poses an excellent study system to investigate whether increases in resource availability lead to an increase in plant species richness. Most sunlight is absorbed by the species-poor tree canopy, making the much more species-rich understorey species inhabit a severely resource-limited habitat. Additionally, the heterogeneity of light availability, resulting from management-moderated tree composition and age structure, may contribute to species coexistence. One would therefore expect that the diversity in the herb layer correlates positively with either the overall light availability, or the light heterogeneity, depending on whether resource availability or heterogeneity are more important drivers of diversity. To test this idea, we assessed variability of light conditions in 75 forest plots across three ecoregions with four different methods.

**Results:**

We correlated these data with vegetation relevés and found light availability to be strongly positively correlated with understorey plant species richness, as well as with understorey cover. Light variability (assessed with two approaches) within plots was positively correlated with transmittance, but did not improve the relationship further, suggesting that the main driver of species richness in this system is the overall resource availability. Two of the three beech-dominated regions exhibited near-identical effects of light transmittance, while the third, featuring pine alongside beech and thus with the longest gradient of transmittance and lowest species richness, displayed a weaker light response.

**Conclusions:**

While site conditions are certainly responsible for the trees selected by foresters, for the resulting forest structure, and for the differences in plant species pools, our results suggest that light transmittance is a strong mediating factor of understorey plant species richness.

## Introduction

The question of what determines local species diversity has puzzled ecologists for a long time. Ecological theory has made various conjectures about driving factors. One is the idea that more resources allow a greater number of individuals to coexist, as well possibly allowing for lower specialization, thus leading to higher diversity. Another idea, closely connected to niche-theory and not mutually exclusive with the previous [1], is that species are specialized to perform best at particular resource levels, and thus a greater diversity in local resource conditions should allow a greater local species richness to emerge [2].

Understorey forest vegetation provides a nice test case for these ideas, owing to the particularly simple and well-defined resource conditions in this ecosystem. Although nutrients and water are important, light, due to its essential role for plant production, is most likely the most limiting resource for plants in the understory layer [3–5]. The tree growth form is the ultimate result of competition for light, and dark forest floor the inevitable consequence, in both managed and natural forests [6]. According to the previous exposition, we would expect that increasing light availability could directly affect diversity by increasing resource levels [7], or indirectly via increasing the density of individuals, and more individuals mean more species [8, 9]. Moreover, we would also expect that light heterogeneity is positively correlated to species richness, due to specialization of species on particular environments.

The empirical evidence for either of these correlations is mixed. Studies have not always found a relationship between light availability and plant species richness (e.g. [10]), but for forest understorey vegetation, a decrease of light availability in forest succession, and a concurrent reduction in species richness was often observed [11–13]. Few of these studies, however, record both average light and light heterogeneity. As both variables tend to be correlated, it is not clear whether their effects were clearly separated, and it may thus well be the heterogeneity of light conditions, rather than availability per se, which drives understorey plant species richness [14].

The issue is complicated by methodological challenges with quantifying understorey light availability. The most common method measures canopy closure (or its inverse, canopy openness), typically computed from hemispheric photographs or visual assessment [15]. While this approach may match well subjective estimates of light regimes by forest scientists [16], and is able to reflect broad light gradients within a forest, e.g. after selective logging [17], it is long known [18] to correlate poorly with light availability measured near the ground by photosynthetically active radiation (PAR) sensors (e.g. [19–21]). One reason is that counting the sky-pixels in a hemispheric photograph is affected by both the camera’s resolution and the algorithm used by the software. Another is that the relative amount of indirect light is underestimated by hemispheric photographs unless explicitly measured as diffuse non-interceptance [21]. As a consequence, 80% canopy cover by hemisphere yields very different light availability at the forest floor in needle and broadleaf forests, for example. Similarly, neither gap sizes nor Ellenberg light indicator values seem to be consistently good predictors of transmittance (e.g. [22–24], but see [25] for regional adjustment of indicators to lidar measurements). Hence, we regard the direct measurement of (photosynthetically active) light availability at the forest floor as the currently only uncontroversial way to assess light conditions. Moreover, direct local measurements allow a much better assessment of light heterogeneity than hemispheric photographs, although multi-location photographs could in principle be used at high spatial resolution as well. Recently, high-resolution remote sensing data and terrestrial laser scanning of canopy closure were shown to be poorly related to modelled light availability [22]. However, we found no reference to validate remote sensing-based light transmittance prediction to the understorey with field measurements (encouragingly, Peng et al. [26] show a very high correlation between modelled, LiDAR-based light availability and measured across-the-spectrum radiation at the forest floor).

Indeed, a thorough literature search for the relation between directly assessed light availability and understorey plant species richness in temperate forests, scanning the abstracts of over 900 papers, only identified five such studies (see Additional file 1 for details). Of these, two studies report an increase of species richness with light transmittance [13, 27], two no response [28, 29] and one a decrease of species richness ([23], reporting Shannon diversity rather than species richness). It must be noted that our review did not unearth possibly relevant parts of the grey literature, such as early work of [30, 31], although at that time direct light measurements were extremely rare (reviewed in [5]).

A further issue for the analysis is that there are various other factors that affect both species richness and light conditions, and may thus confound an analysis of their correlation. Among those, forest management, in itself strongly dependent on soil type, pH and topography, is particularly important. A meta-analysis [32] found that selection harvests have a positive effect on understorey species richness when compared to regeneration harvests (i.e. clearcut, shelterwood), although no difference in species richness was observed when just comparing harvested to unharvested stands in general (in line with the findings of [33], but in contrast to [34]). In general, many studies find effects of management on understory diversity, and management clearly also affects light available for forest understorey plants. Prévost and Raymond [17], for example, found that two to three times as much PAR reached the forest floor in 20–30 m gaps as opposed to uncut forest.

The problem of confounding between management and light, however, goes the other way around as well. Metrics of forest structure, reviewed in their role as potential predictors for biodiversity in [35], rely on a wide range of field measurements such as DBH-distributions and bark types, but seem to completely overlook the easy-to-assess light transmittance. Tellingly, even Röhrig and Ulrich’s [36] monography of Temperate Deciduous Forests largely ignores light as a resource and indeed does not even list the word “light” in its index, and Currie and Bergen’s [37] more recent treatment is no different. This discrepancy between the general appreciation and understanding of the importance of light for temperate forest vegetation and its actual assessment in field studies prompted our study.

Specifically, we examine whether light transmittance and its spatial variability has consistent effects on understorey plant species richness across different temperate forest types. We quantify light transmission in four different ways, assessing alternative facets of light availability. More specifically, we ask the following questions:

1. How does understorey plant species richness relate to light availability at the ground floor?
2. Does spatial variability of light conditions contribute to explaining plant species richness?
3. How do light measurements correlate among methods?
4. How does understorey plant cover relate to light availability, and, again, which quantification is correlated best with it?
5. Does plant cover determine plant species richness, along the lines of the more individuals hypothesis, or does it respond in tandem to light availability?

## Results

### Interrelations of light measurements

The four approaches to quantifying light availability were moderately well correlated (|r| < 0.66, see Table 1). Crown projection area exhibited the lowest correlations with the other measurements (|r| < 0.34).

**Table 1:** Correlations among approaches to measure light availability in the understorey

	Transmittance	Canopy cover	Openness	Crown projection
Transmittance	–	− *0.56*	*0.61*	− *0.38*
*Canopy cover*	− *0.54*	–	− *0.66*	*0.42*
*Openness*	*0.66*	− *0.59*	–	− *0.33*
*Crown projection*	− 0.15	*0.34*	− 0.20	–

Upper triangle of the correlation matrix are Spearman’s correlation coefficients, lower Pearson’s. Sstatistically significant (p < 0.05) correlations are printed in italic

### Plant species richness responses to light

**Fig. 1:**
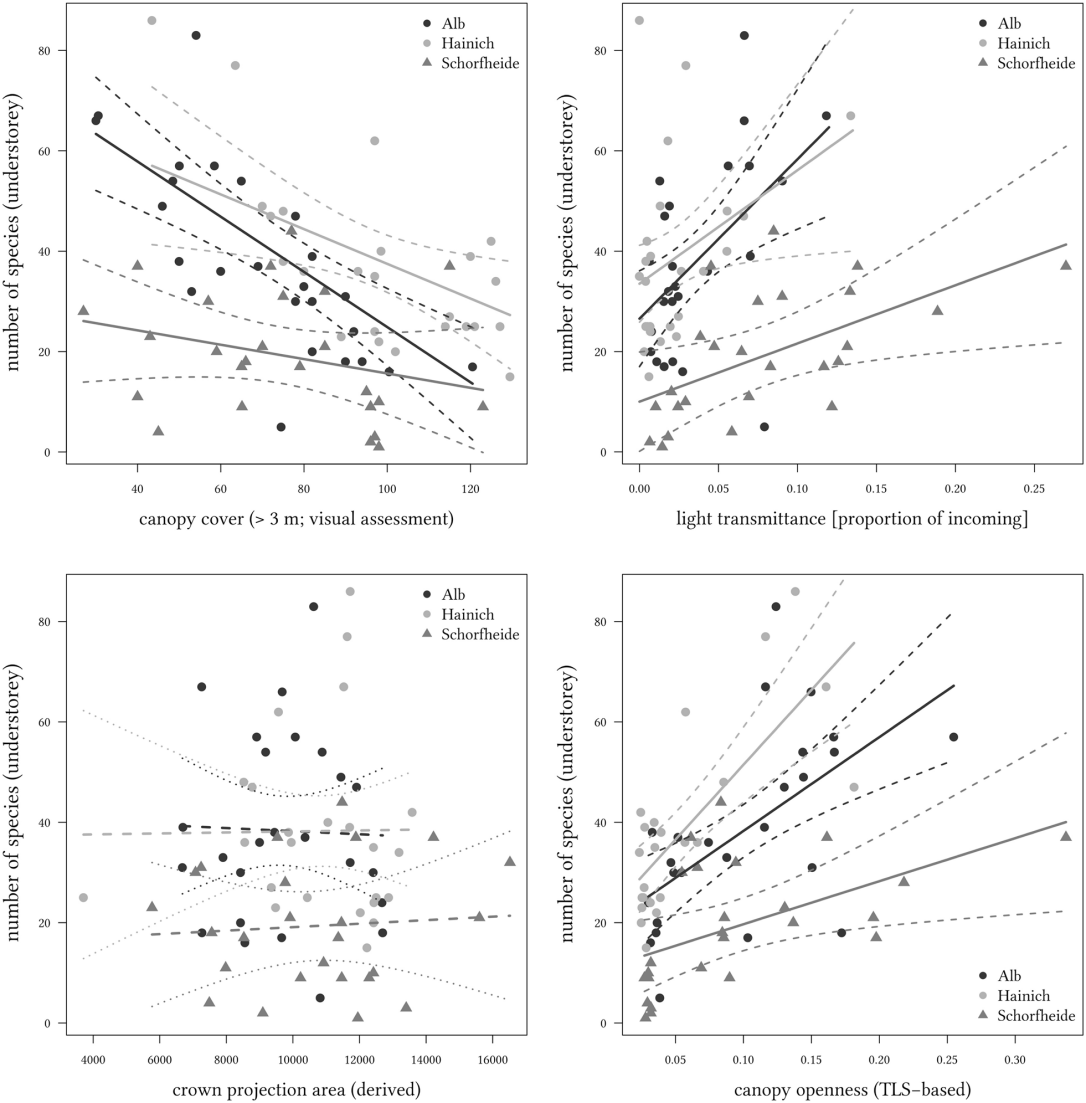
Effect of light availability on the number of vascular plant species (per 400 m^2^) in the forest understorey. Depicted are the four different ways to quantify light availability: direct measurement (top left); estimated cover of canopy > 5 m (top right); hemisphere simulation based on terrestrial laser scanning data (bottom left); and crown projection area derived from typical crown sizes per species and their position in the plots (bottom right). Dashed lines indicate 95% confidence interval of the regression. Non-significant regressions are indicated by dashed lines (and dotted confidence lines)

Three of the four approaches to quantifying light availability in the understorey were significantly related to understorey plant species richness (Table 2), with crown projection area being the exception. In two cases, canopy cover and openness, an interaction with region was observed also. Observer estimated canopy cover displayed the closest relation with species richness (model R^2^_adj_ = 0.55), followed closely by TLS-based openness (R^2^_adj_ = 0.52), and directly measured transmittance (R^2^_adj_ = 0.36), while crown projection was without predictive value (Fig. 1). The three regions differed substantially in average soil, climate, management and species pool size, yet forests on luvisols (Hainich) showed a near-identical response to increased light availability to those on leptosols and cambisols (Alb). In contrast, dry, sandy soils (Schorfheide) supported a lower number of plant species, which responded less strongly to increasing light availability (Fig. 1).

**Table 2:** ANOVA table of the effects of region and light availability on understorey plant species richness

	Effect	DF	SS	*F*
Transmittance	Region	2	6133	13.5***
	Light	1	3812	16.8***
	Region × light	2	837	0.166
	Residuals	68	15,657	R^2^_adj_=0.36
Canopy cover	Region	2	5734	18.3***
	Light	1	6791	43.4***
	Region × light	2	2039	6.52**
	Residuals	68	10,641	R^2^_adj_=0.55
Openness	Region	2	5955	17.2***
	Light	1	6665	38.6***
	Region × light	2	1778	5.15**
	Residuals	68	11,852	R^2^_adj_=0.52
Crown projection	Region	2	5954	10.0***
	Light	1	6	0.0203
	Region × light	2	23	0.402
	Residuals	68	20,165	R^2^_adj_=0.17

Significant effects are indicated by asterisks behind F-values (*** and ** indicated P < 0.001 and 0.01, respectively). Model fit is indicated by adjusted R^2^. Total sum of squares is the same for all models (26,148), and effects are thus directly comparable across models

### Light heterogeneity

For transmittance and openness, we also have available measures of spatial heterogeneity of light availability. In both cases, the standard deviation was highly correlated with the mean (transmittance: *r* = 0.82; openness: *r* = 0.65). Adding log(sd(transmittance)) to the model of Table 2 yielded a significantly *negative* effect of light heterogeneity (F_1, 67_ = 4.43, p < 0.05) and improved the model to an R^2^_adj_ = 0.42, thus providing some evidence *against* the expected positive effect of light heterogeneity on plant species richness. In contrast, adding the standard deviation of openness to the model of Table 2 neither increased its fit, nor did it yield a significant effect of light heterogeneity (neither as linear nor as log-transformed predictor; F_1, 67_ = 2.12, p = 0.15, R^2^_adj_ = 0.55). Using the coefficient of variance instead of the standard deviation showed the same pattern but lower model fits (not shown).

### Understorey plant cover response to light

Herbaceous cover was well predicted (R^2^_adj_ = 0.69) by a combination of region, openness and canopy cover and a canopy cover × region interaction (for ANOVA table and figure see Additional file 1). Openness had a positive and canopy cover a negative effect, with openness being substantially more important (partial R^2^ = 0.28 vs 0.05, respectively).

### Effect of plant cover on plant species richness

Herbaceous cover was highly correlated with understorey plant species richness (*r* = 0.71). Adding the herbaceous cover to the best model of Table 2, the one using canopy cover as predictor, yielded an improvement of around 0.06 units to an R^2^_adj_ = 0.61. The contribution of cover was partial R^2^ = 0.10, taken some of the explanatory power off region and the region-canopy cover interaction. This suggests that the effect of light on plant species richness was only to a limited degree mediated by plant cover.

## Discussion

In this study, we set out to test the effect and relative importance of light availability, and light heterogeneity, on plant species richness in the understorey of three temperate forest ecosystems. Our main findings are that plant species richness increased with the proportion of light that reaches the forest floor; that only a small part of this effect is probably mediated by the effect of light on plant abundance; and that light heterogeneity is much less important for species richness than light availability as such.

Our results regarding a positive effect of light on richness are in line with some previous studies that directly measured both understorey plant species richness and light availability [13, 27]. Although qualitatively anticipated, our results revealed an unexpectedly strong and consistent effect of light availability in the forests of three regions, despite very different soil and climatic conditions. Moreover, the lack of any effect of light heterogeneity is remarkable and in contrast to reports from the tropics [44]. Instead, and in line with some previous studies [38, 39], species richness correlated highly with understorey vegetation cover (see Additional file 1), *prima facie* suggesting that the more-individuals-hypothesis may hold in this system [9]. However, in a combined model, vegetation cover explained only a small part of the variability in species richness, while canopy cover was still far more important.

Our literature review revealed that only a handful of studies *directly* measured both light and understory species richness in temperate forests. It also showed that the majority of studies rely on proxies of transmittance such as canopy closure. However, even direct measurements of light transmittance can be biased by several factors: for instance, light conditions in spring will be different from those in summer, and a single day is arguably problematic as proxy for the entire season’s light effects on understorey plant growth. For this reason we were positively surprised by the strong correlation between our one-off summer light transmittance data and plant species richness. Late summer does represent the light conditions for the majority of the growing season, from the end of foliation (in late April) to leaf shedding (in October). Spring geophytes contribute just over 10 species to the total species richness, so we believe that our peak-season measurements are providing a good representation of transmittance for the largest part of the species over most of the vegetation period. Continuous light measurements would be more precise, but are also much costlier (e.g. repeated monthly measurements during growing season: [23]).

One of the potential reasons for the positive effect of light on plant species richness is that very few species in these communities are actually adapted to very low light conditions (hygromorphic species such as *Impatiens noli*-*tangere* or *Paris quadrifolia*) and would disappear under higher transmittance. Or in other words, the majority of species in our forests are shade tolerant, rather than shade loving, and seem to be thriving even at only 10% of open land PAR. As we did not investigate forest gaps or edges but rather the forest interior, our findings thus complement the literature on gap and edge effects on plant species richness (e.g. [45–48]).

The best predictor of understorey plant species richness was estimated canopy cover above 5 m height. It was assessed by the same people who did the vegetation relevés, in exactly the same place and on the same day, thereby yielding a high consistency of both data sets. Similar to the direct light measurements, it only represents a single snapshot in summer, but one that may subjectively include more impressions than only the light availability at the forest floor. While rapid and working well, for our purpose, it is much more difficult to standardise and reproduce.

An alternative approach to light assessment may be high-resolution laser scanning data, either by aerial or terrestrial scanning [45]. In fact, Ehbrecht et al. [49], whose terrestrial laser scanning data were used to derive the canopy openness used in the present study, show that a much higher resolution can be achieved than typically feasible with hemispheric photographs. It is thus encouraging to see that their data are so predictive of forest understorey richness.

The fourth approach, based on the position and dimension of each tree in the plot and typical size-dependent crown sizes, only poorly predicted understorey plant species richness (Table 2) and correlated least with other methods (Table 1). The variability of tree crown makes it difficult to derive universally applicable crown shapes, hence this database-derived measures seem less suitable to describe in-site conditions of light availability.

Light conditions are affected by a variety of causes [50, 51], with soil and climate conditions providing the background for tree species selection by foresters. Hence the dry, sandy soils of Schorfheide are dominated by Scots pine (*Pinus sylvestris*) and, to a lesser extent, beech; the heavy, wet soils of Hainich by European beech (*Fagus sylvatica*); and the warm, calcareous soils of Alb by beech and Norway spruce (*Picea abies*) [38, 41, 52]. Within each region, management ranged from unmanaged forest to age class forest, contributing to the gradient of light intensities we observed.

Across the 16 studies reporting a relationship of species richness and light conditions at all (i.e. including indirect measures), light conditions were often confounded with tree species, temperature at forest floor, soil properties, successional stage and management [19, 23, 53]. This is most certainly also the case for the plots in our analysis (see [1]; also [54], who worked on the same study plots). Many of these factors could again affect species richness, and thus it is possible that parts of the light effects that we report here are actually indirect and mediated through other variables, or that other variables affect species richness independent of light. For practical field work, we therefore stress that by no means would we suggest that only light is important, but rather that light should be measured routinely in the same way as soil nutrients or soil moisture, to do its potential influence more justice. Teasing apart the different causal pathways between soil, climate, management, light and vascular plant species richness remains an open challenge, particularly with the limited number of data points in each region.

In conclusion, the strong relationship between light availability at the forest floor and its species richness commends itself as important causal link between forest structure and an important element of its diversity. Measuring light directly in the field on a one-off basis does not seem to provide an improvement over traditional expert guesstimates of canopy closure or indeed modern terrestrial laser scanning approaches.

## Methods

### Study sites

Our study sites are part of the Biodiversity Exploratories (BEs, http://www.biodiversity-exploratories.de, [38]). The Biodiversity Exploratories comprise a set of standardized temperate forest plots covering a range of management types in three different regions of Germany: (1) Biosphere reserve Schorfheide-Chorin located in the lowlands of NE Germany (3–140 m a.s.l.), (2) National Park Hainich-Dün located in the hilly Central Germany (285–500 m a.s.l.), and (3) Biosphere reserve Swabian Jura, a low mountain range in SW Germany (460–860 m a.s.l.).

Schorfheide-Chorin, as a glacially formed lowland region, is the driest of the three regions with an annual precipitation of 520–580 mm at a mean annual temperature of around 8 °C. The soils developed from a glacial till, are relatively sandy and variable at small scales, with Dystric Cambisols being the dominant forest soil type. Its forests are dominated by pine and beech. 143 vascular plant taxa were recorded for the 25 plots used in this analysis. Hainich-Dün is slightly wetter (600–800 mm) and colder (7.5 °C) but differs more substantially in its geology and soils. The triassic limestone is covered by loess, leading to Eutric Cambisols and luvisols, with strong variation in soil depth. The soil texture is mainly silty, loamy or clayey and the forests are dominated by beech with a lower and variable share of other deciduous species, supporting 177 plant taxa. The Swabian Jura is a low mountain range of calcareous bedrock, with soils being dominated by Leptosols and Eutric Cambisols. It receives 700–1000 mm of rain per year and annual mean temperature is 6–7 °C. The clayey soils support forests rich in beech and spruce, with the highest species richness of the three regions at 194 plant taxa. A more detailed description is provided by Fischer et al. [38] for the exploratory regions and Schall et al. [34] for its forests.

### Field measurements

We assessed light availability in four different way: direct measurement, estimating canopy cover by eye, reconstruction of canopy openness from terrestrial laser scanning, and computation of crown projection area from crown shapes and tree positions. Measurements were carried out on 25 mature forest plots in each of the three Biodiversity Exploratories. Regrettably not all types of light measurements could be taken in the same year, but plot management was limited to a few plots and to sub-canopy thinning treatments during the data collection period (see Additional file 1).

For direct light measurements, we recorded absolute values of photosynthetically active radiation (PAR in µmol m^−2^ s^−1^) below the canopy at breast height. Measurements were taken on 10 days in August and September 2017. In each 100 m × 100 m forest plot, we recorded absolute values of photosynthetic active radiation (PAR, in µmol m^−2^s^−1^) below the canopy at breast height, using the Li-COR 191R Line Quantum sensor, Li-COR Inc., Lincoln, Nebraska), at 25 random locations covering the entire surface of the plot, and at regular intervals of 30 s for a total duration of 15 min. For each plot, we additionally recorded light on the nearest clearing or canopy gap as a reference (using Li-COR’s Quantum 190R sensor). We computed light availability as transmittance, i.e. the proportion of available photosynthetically active radiation (obtained from reference measurements) reaching the forest floor, thereby correcting for different absolute radiance across the days of the campaign. Mean and standard deviation of these measurements were calculated for each plot. Data from one plot were lost due to computer failure.

Percentage canopy cover was estimated in a subplot of 20 × 20 m on which also the vegetation relevés were taken (see below). Cover of trees was estimated for two strata, those smaller and taller than 10 m by the same people who did the vegetation relevés. We use the sum of the two strata as canopy cover, which may hence exceed 100%.

Canopy openness was computed from terrestrial laser scanning data (for details see [39]). A Faro Focus 3D 120 (Faro Technologies Inc., Lake Mary, USA) laser scanner employing phase-shift technology scanned a field of view of 90° (step width of 0.14°, range limit 120 m), resulting in a hemisphere 3.25 Mio laser beams. The scanner was set up, in summer 2014, in nine regularly spaced positions over 100 × 100 m in each plot. From this point cloud, a hemispherical image (60° opening angle) was simulated and the cover of sky quantified.

Finally, crown projection areas were computed for each plot based on field-inventory data recorded between 2015 and 2016 [34]. Using species-specific parameters [40], the crown size of each tree growing in the plot was estimated from its diameter, and summed, yielding the unitless crown area (i.e. 10 000 m^2^ per ha). Due to layering and uniform shapes employed, crown projection can be larger than 1.

Percentage cover estimates of each understorey vascular plant species was quantified in spring and summer 2017 within subplots of 20 m × 20 m. To assess the species richness and correct the cover values of plant species per plot, we combined the spring and summer records, using the higher cover value for each species (for details see [41]). All data are available from BExIS (http://www.bexis.uni-jena.de, data sets 22506, 22766, 23686 and 25146).

### Statistical analysis

All analyses were conducted with R version 3.5.1 [42]. We analysed the effect of light transmission on understorey species richness and their cover using a Generalised Additive Model (R package mgcv, [43]) with region as factor. As the GAM indicated that the effects of transmittance and standard deviation of transmittance were linear, we continued all further analyses with a GLM with species richness as the response and predictors including light, region and an interaction between region and light, respectively. This model was modified for the effect of standard deviation of light availability or by including several measures of light availability simultaneously.

## Supplementary information

**Additional file 1.** Additional information and analyses.

## Data Availability

Access to data is available through the project database (https://www.bexis.uni-jena.de, data sets 22506, 22766, 23686 and 25146). R-code for analysis is available on request from the first author.

## References

[1] Cardinale BJ, Hillebrand H, Harpole WS, Gross K, Ptacnik R (2009). Separating the influence of resource ‘availability’ from resource ‘imbalance’ on productivity–diversity relationships. Ecol Lett.

[2] Chesson P (2000). Mechanisms of maintenance of species diversity. Annu Rev Ecol Syst.

[3] Cornwell WK, Grubb PJ (2003). Regional and local patterns in plant species richness with respect to resource availability. Oikos.

[4] Crawford RMM (1989). Studies in plant survival: ecological case histories of plant adaptation to adversity.

[5] Eber W (1972). Über das Lichtklima von Wäldern bei Göttingen und seinen Einfluß auf die Bodenvegetation. Scr Geobot..

[6] Beaudet M, Messier C, Leduc A (2004). Understorey light profiles in temperate deciduous forests: recovery process following selection cutting. J Ecol.

[7] Onoda Y, Saluñga JB, Akutsu K, Aiba S, Yahara T, Anten NPR (2014). Trade-off between light interception efficiency and light use efficiency: implications for species coexistence in one-sided light competition. J Ecol.

[8] Stevens MHH, Carson W (1999). Plant density determines species richness along an experimental fertility gradient. Ecology.

[9] Storch D, Bohdalková E, Okie J (2018). The more-individuals hypothesis revisited: the role of community abundance in species richness regulation and the productivity-diversity relationship. Ecol Lett.

[10] Adler PB, Seabloom EW, Borer ET, Hillebrand H, Hautier Y, Hector A (2011). Productivity is a poor predictor of plant species richness. Science.

[11] Bazzaz FA (1979). The physiological ecology of plant succession. Annu Rev Ecol Syst.

[12] Connell JH, Slayter RO (1977). Mechanisms of succession in natural communities and their role in community stability and organization. Am Nat.

[13] Lichter J (1998). Primary succession and forest development on coastal Lake Michigan sand dunes. Ecol Monogr.

[14] Bartels SF, Chen HYH (2010). Is understory plant species diversity driven by resource quantity or resource heterogeneity?. Ecology.

[15] Lieffers VJ, Messier C, Stadt KJ, Gendron F, Comeau PG (1999). Predicting and managing light in the understory of boreal forests. Can J For Res.

[16] Anderson MC (1964). Light relations of terrestrial plant communities and their measurement. Biol Rev.

[17] Prévost M, Raymond P (2012). Effect of gap size, aspect and slope on available light and soil temperature after patch-selection cutting in yellow birch–conifer stands, Quebec, Canada. For Ecol Manag..

[18] Madgwick HAI, Brumfield GL (1969). The use of hemispherical photographs to assess light climate in the forest. J Ecol.

[19] Brown MJ, Parker GG (1994). Canopy light transmittance in a chronosequence of mixed-species deciduous forests. Can J For Res.

[20] Engelbrecht BMJ, Herz HM (2001). Evaluation of different methods to estimate understorey light conditions in tropical forests. J Trop Ecol.

[21] Tinya F, Mihók B, Márialigeti S, Mag ZS, Ódor P (2009). A comparison of three indirect methods for estimating understory light at different spatial scales in temperate mixed forests. Community Ecol..

[22] Alexander C, Moeslund JE, Bøcher PK, Arge L, Svenning J-C (2013). Airborne laser scanner (LiDAR) proxies for understory light conditions. Remote Sens Environ.

[23] Fuxai X, Fousseni F, Chungang P, Huaijiang H, Xiuhai Z (2014). Effect of overstory on the seasonal variability of understory herbs in primary broad-leaved Korean pine forest of Changbai Mountain. Afr J Biotechnol..

[24] Szymura TH, Szymura M, Macioł A (2014). Bioindication with Ellenberg’s indicator values: a comparison with measured parameters in Central European oak forests. Ecol Indic..

[25] Zellweger F, Baltensweiler A, Schleppi P, Huber M, Küchler M, Ginzler C (2019). Estimating below-canopy light regimes using airborne laser scanning: an application to plant community analysis. Ecol Evol..

[26] Peng S, Zhao C, Xu Z (2014). Modeling spatiotemporal patterns of understory light intensity using airborne laser scanner (LiDAR). ISPRS J Photogramm Remote Sens..

[27] Márialigeti S, Tinya F, Bidló A, Ódor P (2016). Environmental drivers of the composition and diversity of the herb layer in mixed temperate forests in Hungary. Plant Ecol.

[28] Houle G (2007). Determinants of fine-scale plant species richness in a deciduous forest of northeastern North America. J Veg Sci.

[29] Bartels SF, Chen HYH (2013). Interactions between overstorey and understorey vegetation along an overstorey compositional gradient. J Veg Sci.

[30] Daxer H (1934). Über die Assimilationsökologie der Waldbodenflora. Jahresbücher Für Wiss Bot..

[31] Knapp R (1960). Licht und Arten-Zusammensetzung in Wald- und Strauchgesellschaften in hohen Lagen und im Bereich der Baumgrenze. Wetter Leben..

[32] Duguid MC, Ashton MS (2013). A meta-analysis of the effect of forest management for timber on understory plant species diversity in temperate forests. For Ecol Manag..

[33] Paillet Y, Bergès L, HjÄltén J, Ódor P, Avon C, Bernhardt-Römermann M (2010). Biodiversity differences between managed and unmanaged forests: meta-analysis of species richness in Europe. Conserv Biol.

[34] Schall P, Schulze E-D, Fischer M, Ayasse M, Ammer C (2018). Relations between forest management, stand structure and productivity across different types of Central European forests. Basic Appl Ecol.

[35] Storch F, Dormann CF, Bauhus J (2018). Quantifying forest structural diversity based on large-scale inventory data: a new approach to support biodiversity monitoring. For Ecosyst..

[36] Röhrig E, Ulrich B. Temperate deciduous forests. Amsterdam: Elsevier; 1991. schnei.

[37] Currie WS, Bergen KM, Jørgensen SE (2009). Temperate Forest. Ecosystem Ecology.

[38] Fischer M, Bossdorf O, Gockel S, Hänsel F, Hemp A, Hessenmöller D (2010). Implementing large-scale and long-term functional biodiversity research: the biodiversity exploratories. Basic Appl Ecol.

[39] Ehbrecht M, Schall P, Juchheim J, Ammer C, Seidel D (2016). Effective number of layers: a new measure for quantifying three-dimensional stand structure based on sampling with terrestrial LiDAR. For Ecol Manag..

[40] Riedel T, Henning P, Polley H, Schmitz F, Schwitzgebel F (2017). Die dritte Bundeswaldinventur (BWI 2012).

[41] Boch S, Prati D, Müller J, Socher S, Baumbach H, Buscot F (2013). High plant species richness indicates management-related disturbances rather than the conservation status of forests. Basic Appl Ecol.

[42] R Core Team. R: A Language and Environment for Statistical Computing. Vienna, Austria. http://www.R-project.org: R Foundation for Statistical Computing; 2019.

[43] Wood SN (2017). Generalized additive models: an introduction with R.

[44] Watling JR, Press MC. Light heterogeneity in tropical rain forests: photosynthetic responses and their ecological consequences. In: The Ecological consequences of environmental heterogeneity. Oxford: Blackwell; 2000. p. 131–53.

[45] Getzin S, Wiegand K, Schöning I (2012). Assessing biodiversity in forests using very high-resolution images and unmanned aerial vehicles: assessing biodiversity in forests. Methods Ecol Evol.

[46] Gonzalez M, Ladet S, Deconchat M, Cabanettes A, Alard D, Balent G (2010). Relative contribution of edge and interior zones to patch size effect on species richness: an example for woody plants. For Ecol Manag..

[47] Raghubanshi AS, Tripathi A (2009). Effect of disturbance, habitat fragmentation and alien invasive plants on floral diversity in dry tropical forests of Vindhyan highland: a review. Trop Ecol..

[48] Schnitzer SA, Carson WP (2001). Treefall gaps and the maintenance of species diversity in a tropical forest. Ecology.

[49] Ehbrecht M, Schall P, Ammer C, Fischer M, Seidel D (2019). Effects of structural heterogeneity on the diurnal temperature range in temperate forest ecosystems. For Ecol Manag..

[50] Barbier S, Gosselin F, Balandier P (2008). Influence of tree species on understory vegetation diversity and mechanisms involved—a critical review for temperate and boreal forests. For Ecol Manag..

[51] Bartemucci P, Messier C, Canham CD (2006). Overstory influences on light attenuation patterns and understory plant community diversity and composition in southern boreal forests of Quebec. Can J For Res.

[52] Schall P, Gossner MM, Heinrichs S, Fischer M, Boch S, Prati D (2018). The impact of even-aged and uneven-aged forest management on regional biodiversity of multiple taxa in European beech forests. J Appl Ecol.

[53] Härdtle W, von Oheimb G, Westphal C (2003). The effects of light and soil conditions on the species richness of the ground vegetation of deciduous forests in northern Germany (Schleswig-Holstein). For Ecol Manag..

[54] Heinrichs S, Ammer C, Mund M, Boch S, Budde S, Fischer M (2019). Landscape-scale mixtures of tree species are more effective than stand-scale mixtures for biodiversity of vascular plants, bryophytes and lichens. Forests..

