# Supplementary material for: Plant species richness increases with light availability, but not variability, in temperate forests understorey
Source: BMC Ecol. 2020 Jul 29;20:43. doi: 10.1186/s12898-020-00311-9 (PMC7392730; doi:10.1186/s12898-020-00311-9)
Supplement: Supplementary file 1 — Additional file 1. Additional information and analyses. [file 12898_2020_311_MOESM1_ESM.docx]

Additional Material

# S1. Literature review

We conducted a systematic literature search for studies investigating the effect of light on understorey plant species richness in temperate and boreal forests. In January 2019, we searched the Web of Science core collection using the search string

‘(species (diversity OR richness OR number OR composition OR community OR assemblage) OR abundance OR diversity) AND (forest) AND (light OR "canopy gap" OR "open canopy" OR "closed canopy" OR "canopy cover" OR "canopy closure" OR sun OR shade OR transmission OR reflectance OR absorption OR irradian* OR illumination OR radiation OR par) NOT tropical AND “herb OR forb” ’.

Both “vascular plant” and “understor(e)y” inflated the results by studies investigating primarily forest fauna, therefore the more restrictive term “herb OR forb” was used.

This search yielded 914 hits (see separate file), which were then scanned for providing an analysis of understorey plant species richness and some measure of light climate in the understorey. Only 28 studies matched this criterion (see supplementary material), 16 actually providing information on the light-richness relationship, and only five studies measure light transmittance in the field. We acknowledge that perusing this large number of abstracts may have led to overlooking some relevant studies.

## S2. The 27 studies that remained after focus on (a) measuring plant species richness and (b) light availability; with comments regarding their findings and inclusion/exclusion from further discussion

Direct light measurements (comments in bold):

*positive effect on S*: Lichter 1998; Marialigeti et al. 2016

*negative effect on S*: Fuxai et al. 2012

*no effect on S*: Houle 2007; Bartels & Chen 2013

Hemisphere, canopy openness, gap size, etc.:

*no effect, mixed effects or not reported*: Axamnova et al. 2011, 2013; Borchsenius et al. 2004, Bruelheide & Luginbuehl 2009, Wulf & Naaf 2009

*positive effect on S*: Hofmeister et al. 2009

*negative effect on S*: North et al. 2005, Plue et al. 2013, Tan et al. 2016

Alberdi, M., et al. (1988). "Light intensities and energy content of plant commjunities in the Andes of south Central Chile." Turrialba 38(4): 323-331. [source could not be found]

Ampoorter, E., et al. (2015). "Disentangling tree species identity and richness effects on the herb layer: first results from a German tree diversity experiment." Journal of Vegetation Science 26(4): 742-755. [no light-S analysis]

Axmanova, I., et al. (2013). "Plant species richness-productivity relationships in a low-productive boreal region." Plant Ecology 214(2): 207-219. [light from hemisphere photographs and computer, not measured; no effect of absolute direct or diffuse light on richness]

Axmanova, I., et al. (2012). "The species richness-productivity relationship in the herb layer of European deciduous forests." Global Ecology and Biogeography 21(6): 657-667. [canopy cover, no light measurements]

Axmanova, I., et al. (2011). "Environmental factors influencing herb layer productivity in Central European oak forests: insights from soil and biomass analyses and a phytometer experiment." Plant and Soil 342(1-2): 183-194. [light from hemisphere photographs and computer, not measured; no effect of light on S]

Bartels, S. F. and H. Y. H. Chen (2013). "Interactions between overstorey and understorey vegetation along an overstorey compositional gradient." Journal of Vegetation Science 24(3): 543-552. **[no effect of light on S, only on herb cover (Table 4)]**

Borchsenius, F., et al. (2004). "Vegetation structure and diversity of an ancient temperate deciduous forest in SW Denmark." Plant Ecology 175(1): 121-135. [effect of light (direction unclear) on S, confounded with pH and stem density (PCA)]

Brudvig, L. A., et al. (2011). "Dispersal, not Understory Light Competition, Limits Restoration of Iowa Woodland Understory Herbs." Restoration Ecology 19: 24-31. [experimental, no light measurements]

Bruelheide, H. and U. Luginbuehl (2009). "Peeking at ecosystem stability: making use of a natural disturbance experiment to analyze resistance and resilience." Ecology 90(5): 1314-1325. [light affects species composition, no analysis of species richness]

Buckley, D. S., et al. (2003). "Influence of skid trails and haul roads on understory plant richness and composition in managed forest landscapes in Upper Michigan, USA." Forest Ecology and Management 175(1-3): 509-520. [PAR confounded with soil structure (due to logging); no analysis of light effect]

Burton, J. I., et al. (2014). "Experimentally linking disturbance, resources and productivity to diversity in forest ground-layer plant communities." Journal of Ecology 102(6): 1634-1648. [PAR confounded with SWC; no analysis of PAR/SWC vs S]

Burton, J. I., et al. (2009). "Patterns of plant community structure within and among primary and second-growth northern hardwood forest stands." Forest Ecology and Management 258(11): 2556-2568. [light on axis 2, but loadings not shown, nor effects of axes on S]

Calcada, E. A., et al. (2015). "Site productivity overrides competition in explaining the disturbance-diversity relationship in riparian forests." Perspectives in Plant Ecology Evolution and Systematics 17(6): 434-443. [experimental; no analysis of light effects on S]

Dubbert, M., et al. (2014). "Influence of tree cover on herbaceous layer development and carbon and water fluxes in a Portuguese cork-oak woodland." Acta Oecologica-International Journal of Ecology 59: 35-45. [no S measured]

Galhidy, L., et al. (2006). "Effects of gap size and associated changes in light and soil moisture on the understorey vegetation of a Hungarian beech forest." Plant Ecology 183(1): 133-145. [light computed from hemisphere; no analysis of S ~ light]

Gratani, L. (1997). "Canopy structure, vertical radiation profile and photosynthetic function in a *Quercus ilex* evergreen forest." Photosynthetica 33(1): 139-149. [no S]

Härdtle, W., von Oheimb, G., & Westphal, C. (2003). The effects of light and soil conditions on the species richness of the ground vegetation of deciduous forests in northern Germany (Schleswig-Holstein). *Forest Ecology and Management*, *182*(1–3), 327–338. [canopy cover only; no effect on S]

Hofmeister, J., et al. (2009). "The influence of light and nutrient availability on herb layer species richness in oak-dominated forests in central Bohemia." Plant Ecology 205(1): 57-75. [**light computed from hemisphere; non-significant, positive effect of light on S (Fig. 3)**]

Houle, G. (2007). "Determinants of fine-scale plant species richness in a deciduous forest of northeastern North America." Journal of Vegetation Science 18(3): 345-354. [**light measured; no effect on S**]

Jiang, Z. and K. Ma (2015). "Environmental filtering drives herb community composition and functional trait changes across an elevational gradient." Plant Ecology and Evolution 148(3): 301-310. [light measured with camera (“total site factor”); no effect on S reported, only composition (light correlated with temperature, but temperature better predictor of S)]

Lichter, J. (1998). “Primary Succession and Forest Development On coastal Lake Michigan Sand Dunes”. Ecological Monographs 68(4): 487-510. **[light measured (PAR); positive effect on S, but intermediate transmittance best for S (Fig. 12)]**

Marialigeti, S., et al. (2016). "Environmental drivers of the composition and diversity of the herb layer in mixed temperate forests in Hungary." Plant Ecology 217(5): 549-563. [**light measured (36 times per plot); positive effect on S**]

McEwan, R. W. and R. N. Muller (2011). "Dynamics, diversity, and resource gradient relationships in the herbaceous layer of an old-growth Appalachian forest." Plant Ecology 212(7): 1179-1191. [light computed from hemisphere, but used canopy openness; effect only on composition, S not analysed]

North, M., et al. (2005). "Influence of light and soil moisture on Sierran mixed-conifer understory communities." Plant Ecology 177(1): 13-24. [**light computed from hemisphere; more light FEWER herb species (though with CART not really visible**]

Plue, J., et al. (2013). "Forest herb layer response to long-term light deficit along a forest developmental series." Acta Oecologica-International Journal of Ecology 53: 63-72. [light computed; effect of stand age on S, typically associated with lower light]

Tan, Y.-b., et al. (2016). "Effects of canopy structure on understory vegetation in shelterbelt forests along the middle and upper reaches of Pearl River." Shengtaixue Zazhi 35(12): 3148-3156. [**light computed from hemisphere; light and S negatively correlated; abstract only**]

Wulf, M. and T. Naaf (2009). "Herb layer response to broadleaf tree species with different leaf litter quality and canopy structure in temperate forests." Journal of Vegetation Science 20(3): 517-526. [light not measured, only canopy closure; effect on S dependent on canopy species, from negative to slight positive effect]

Fuxai, X., et al. (2012). "Influence of overstory on seasonal variability of understory herbs in primary broad-leaved Korean pine forest of Changbai Mountain." Acta Botanica Boreali-Occidentalia Sinica, 32(2), 370-376. [**light measured; more light → lower Shannon diversity!; also data on both PPFD and gaps (no correlation!)**]

The five studies reporting on direct light measurements and species richness:

Bartels & Chen (2013) report from 51 sites in Quebec and Ontario along a gradient in broadleaf-conifer canopy; they found strong effects of soil characteristics, but no effect of light conditions on shrub and herb richness, despite a positive effect of light on herbaceous cover (in Ontario only). Also in Quebec, Houle (2007) found strong effects of stand density on understorey richness, but that was not mediated by light availability. Fuxai et al. (2014) even report a slight negative effect of photosynthetic photon flux density (PPFD) on herbaceous diversity from a single Korean mixed pine-broadleaf forest site. Interestingly, this study subdivided the site into 1 m^2^ plots, with only a handful of plant species in each. The negative correlation with PPFD may thus be partly explained by the small plot size.

In contrast, Márialigeti et al. (2016), whose 34 plots were most similar to our study both in forest type and plot size, found light conditions to be positively correlated with plant species richness in the understorey, explaining around 20% of the variation, both for all plant species as well as for those deemed to be forest specialists. Lichter (1998) reporting from a coastal dune succession gradient in Michigan, found light penetration to be related to species richness in a hump-shaped fashion, with highest richness at around 50% light penetration.

# 3. Additional analyses

**Figure S1**. Changes in canopy cover (above 5 m) over the study period, as visually estimated in the plots. Management interventions affected only 5 plots to a larger degree. The three regions are colour-coded as in the main text: black: Alb, dark grey: Schorfheide, light grey: Hainich.

**Table S1.** Analysis of variance table for a multiple regression that started with all four measures of light availability as predictor, each in interaction with region. We employed BIC-stepwise model selection.

Response: herbaceous cover

Df Sum Sq Mean Sq F value Pr(>F)

region 2 71831 35915 43.8764 1.003e-12 ***

openness 1 41313 41313 50.4704 1.221e-09 *** CanopyB1B2Cover2017 1 7728 7728 9.4410 0.003115 ** region:CanopyB1B2Cover2017 2 10708 5354 6.5408 0.002602 **

Residuals 64 52388 819

---

Signif. Codes: 0 ‘***’ 0.001 ‘**’ 0.01 ‘*’ 0.05 ‘.’ 0.1 ‘ ’ 1

Coefficients:

Estimate Std. Error t value Pr(>|t|)

(Intercept) 109.32188 24.17239 4.523 2.71e-05 ***

regionH 77.90234 34.29188 2.272 0.02647 *

regionS -122.14977 28.42443 -4.297 6.00e-05 ***

openness_mean 311.83536 66.25547 4.707 1.39e-05 ***

CanopyB1B2Cover2017 -0.96355 0.30738 -3.135 0.00260 **

regionH:CanopyB1B2Cover2017 -0.02698 0.39952 -0.068 0.94636 regionS:CanopyB1B2Cover2017 1.10900 0.38381 2.889 0.00526 **

---

Signif. Codes: 0 ‘***’ 0.001 ‘**’ 0.01 ‘*’ 0.05 ‘.’ 0.1 ‘ ’ 1

Residual standard error: 28.61 on 64 degrees of freedom

Multiple R-squared: 0.7152, Adjusted R-squared: 0.6885

F-statistic: 26.79 on 6 and 64 DF, p-value: 1.028e-15


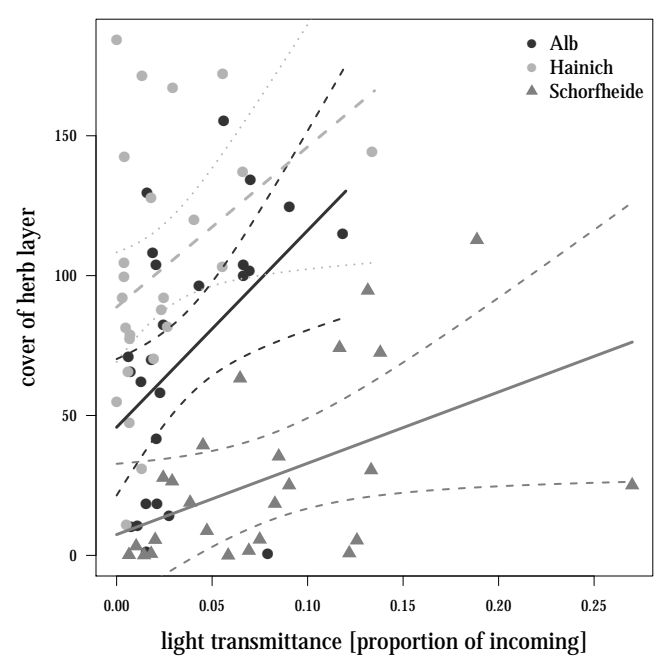


**Figure S2**. Effect of light transmittance on cover of the herbaceous layer (in %).

Figure 2: Effect of light transmittance on the vascular understorey plant cover. Dashed lines indicate 95% confidence interval of the regression. Non-significant regressions are indicated by dashed lines (and dotted confidence lines).
